# Effect of CM15 on Supported Lipid Bilayer Probed by Atomic Force Microscopy

**DOI:** 10.3390/membranes13110864

**Published:** 2023-10-28

**Authors:** Olivia D. Walsh, Leona Choi, Krishna P. Sigdel

**Affiliations:** Department of Physics and Astronomy, California State Polytechnic University, Pomona, CA 91768, USA

**Keywords:** atomic force microscopy, lipid bilayer, antimicrobial peptide, AMP, hybrid peptides, cecropin-A, melittin, bee-venom, peptide–membrane interaction, *E. coli* lipids, CM15

## Abstract

Antimicrobial peptides are key components of the immune system. These peptides affect the membrane in various ways; some form nano-sized pores, while others only produce minor defects. Since these peptides are increasingly important in developing antimicrobial drugs, understanding the mechanism of their interactions with lipid bilayers is critical. Here, using atomic force microscopy (AFM), we investigated the effect of a synthetic hybrid peptide, CM15, on the membrane surface comprising *E. coli* polar lipid extract. Direct imaging of supported lipid bilayers exposed to various concentrations of the peptide revealed significant membrane remodeling. We found that CM15 interacts with supported lipid bilayers and forms membrane-spanning defects very quickly. It is found that CM15 is capable of remodeling both leaflets of the bilayer. For lower CM15 concentrations, punctate void-like defects were observed, some of which re-sealed themselves as a function of time. However, for CM15 concentrations higher than 5 µM, the defects on the bilayers became so widespread that they disrupted the membrane integrity completely. This work enhances the understanding of CM15 interactions with the bacterial lipid bilayer.

## 1. Introduction

Peptides are essential components of a cell. The interactions of peptides with the complex phospholipid bilayer environment play a vital role in the functioning of various membrane-aided cellular processes and the action of the membrane-active antimicrobial peptides [1,2]. Antimicrobial peptides (AMPs) form a critical part of the innate immune system and can be universally found in all domains of life [3]. These peptides can kill bacteria, fungi, and even cancer cells by permeabilizing the cellular membranes. Due to their activity against bacteria, AMPs have been actively tested as potential candidates in the development of antimicrobial agents for the treatment of emerging drug-resistant infections [4,5,6,7,8].

**Figure 1:**
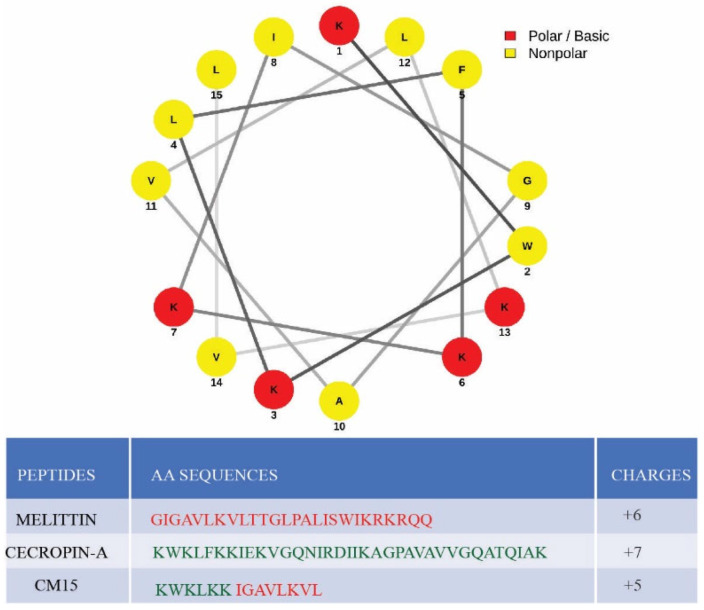
Peptides at a glance. Helical wheel of CM15 peptide that demonstrates the relative positions of amino acids in the peptide along with a table of amino acid sequences of parent peptides and hybrid CM15 peptide. The helical wheel is prepared using reference [15].

Melittin, the bee-venom peptide, is a well-studied example of a lipid-induced folding peptide. It consists of 26 residues (see Figure 1, Table) and possesses a hydrophobic *N*-terminal domain along with a basic *C*-terminal domain. This cytolytic peptide is mostly unstructured while in solution but forms an amphipathic alpha-helix in the presence of lipid membranes and surfactants [9,10]. Melittin exhibits strong broad-spectrum antimicrobial activity and high hemolytic activity towards bacterial and eukaryotic cells [11]. The membrane disruption properties of melittin have been studied using a variety of methods [12]. Membrane-bound melittin can adopt two orientational states; it can either be in an approximate parallel configuration to the membrane surface or be perpendicular to the membrane, forming membrane-spanning pores. Evidence shows that each state is a function of peptide concentration. At low peptide concentrations, melittin binds to the bilayer surface in parallel conformation, which could cause the thinning of the membrane by locally displacing the lipid headgroups [13]. At higher concentrations, the parallel state shifts to a perpendicular state by inserting the peptides into the membrane, leading to the formation of transmembrane pores. At high enough concentration, melittin solubilizes the bilayer [14].

In contrast, cecropin-A is a cationic, 37-residue antimicrobial peptide, initially isolated from silk moth, with a relatively hydrophobic *C*-terminal domain and basic *N*-terminal domain, opposite to that of melittin. It demonstrates a broad antibacterial activity against both gram-negative and gram-positive bacteria. Moreover, it exhibits relatively low toxicity and hemolytic activities toward eukaryotic cells [16]. Similar to melittin, this peptide is mostly unstructured in solution, but it adopts an amphipathic alpha-helix in the presence of organic solvents and lipid membranes [17,18]. Cecropin-A is known to induce the formation of ion-permeable pores in a negatively charged lipid bilayer in the concentration range of 2–5 µM, leading to destabilization and destruction of the bilayer when the concentration exceeds 10 µM [19]. Despite sharing several common features with the melittin peptide, cecropin-A displays surprisingly distinct activities toward bacterial and eukaryotic cells. For example, cecropin-A is found to be more potent than melittin against many multi-drug-resistant gram-negative bacteria [20]. Melittin, cecropin-A, and their derivatives are currently undergoing active investigations as important therapeutic candidates for various human diseases [21,22].

Evolved as a chimeric peptide from melittin and cecropin-A, CM15 has received particular attention due to its broad-spectrum antimicrobial effects coupled with its small size [16]. It comprises the first seven residues of cecropin-A and residues two to nine of melittin. The amino acid composition and arrangements of the peptide are represented via the helical wheel diagram in Figure 1. It features a highly basic *N*-terminal and relatively hydrophobic *C*-terminal domain. CM15 demonstrates similar broad-spectrum antimicrobial activity to cecropin-A, yet lacks the strong hemolytic activity of melittin [17]. Similar to its parent peptides, CM15 displays a random coil structure in solution and exhibits an α-helical secondary structure in the presence of membrane or organic solvents [11,23].

The effects of CM15 interactions with the lipid bilayer have been investigated using a variety of methods, including circular dichroism, sum frequency generation (SFG) spectroscopy [24], fluorescence spectroscopy, UV resonance Raman spectroscopy [25], molecular dynamics simulations [26], patch-clamp techniques [27], site-directed spin labeling EPR [23], DSC, quasistatic light scattering [28], and x-ray diffraction [18]. Previous studies suggest that CM15 affects both bacterial and mammalian model membranes in the form of voltage-independent membrane permeabilization, pore formation, and general membrane disruption [17,18,23,27,28,29,30]. A recent study, conducted at room temperature, revealed that CM15 disrupted the outer leaflet of a bacterial model membrane while having negligible effects on the inner leaflet. The study, however, showed that, at an elevated temperature (~35 ${}^{\circ }$C), CM15 disrupted both leaflets of the membrane [24].

Atomic force microscopy (AFM) has emerged as an important complementary instrument for molecular characterization in biological settings [31,32]. At its core, AFM consists of a micro-robotic arm with a nanoscale force probe that interacts with a sample. The force probe raster scans over a membrane surface to provide real-time high-resolution images of membrane topography in a native physiological buffer environment. This technique allows for direct visualization of membrane defect formation, membrane destruction and conformations, and dynamics of the defects and pores [33,34]. Hence, AMP–lipid systems are well-suited for studies using AFM.

In this work, we employed high-resolution AFM imaging to investigate the lipid bilayer remodeling effects of CM15 for the first time. *E. coli* polar lipid extract, which closely mimics the bacterial cell membrane, was employed. Interestingly, we observed clear effects of CM15 on the supported lipid bilayers at concentrations as low as 0.2 µM. Punctate void-like pores were common at concentrations of 2.5 µM, and large defects leading to complete membrane disruption were observed for 10 µM concentrations. Further, on the timescale of AFM imaging, the peptide produces rapid remodeling.

## 2. Materials and Methods

### 2.1. Materials

Phospholipid was purchased from Avanti Polar Lipids (Alabaster, AL): *E. coli* polar extract (catalog number 100600). All the salts for the buffer solution were purchased from Sigma (St. Louis, MO, USA). Peptide, CM15, was synthesized at GenScript (Piscataway, NJ, USA).

### 2.2. Peptide Solution Preparation

Peptide, CM15, was synthesized (GenScript) with *N*-terminus acetylation and *C*-terminal amidation with purity > 95%. The sequence of CM15 is KWKLFKKIGAVLKVL. The helical wheel of the peptide is shown in Figure 1 and was prepared using reference [15].

The peptide was diluted to the desired concentration in buffer solution (10 mM Hepes pH 7.4, 100 mM KAc, 5 mM Mg (Ac)_2_) prior to AFM imaging.

### 2.3. Lipid Bilayer Preparation

Liposomes were prepared by adopting an established protocol [35]. *E*. *coli* polar extract lipids dissolved in chloroform (Avanti) were blown dry with ultra-high pure nitrogen gas and further dried under vacuum overnight. Dried lipids were suspended in 10 mM Hepes pH 7.4, 100 mM KAc, and 5 mM Mg (Ac)_2_,1 mM EDTA), and were extruded through a polycarbonate membrane (~25 times) with a 100 nm pore diameter (mini-extruder, Avanti) to form large uni-lamellar vesicles (LUVs). The extrusion was performed at room temperature (~25 ${}^{\circ }$C). Supported lipid bilayers were formed by vesicle fusion to freshly cleaved mica surfaces by incubating ~100 µM lipid for ~1 h at room temperature. The supported bilayer was rinsed with 100 µL imaging buffer (10 mM Hepes pH 7.4, 100 mM KAc, 5 mM Mg (Ac)_2_) three times to remove the unattached lipids/lipid bilayers prior to microscopy experiments. The presence of the bilayer was verified either with a height measurement via a surface defect or punch-through measurements of the lipid surface [36,37]. Next, 80 µL of the desired concentration of peptide was injected into the AFM fluid cell for imaging.

### 2.4. AFM Imaging and Analysis

Experiments were performed in imaging buffer (10 mM Hepes pH 7.4, 100 mM KAc, 5 mM Mg (Ac)_2_) at room temperature (~25 ${}^{\circ }$C) using a commercial apparatus (MFP-3D Origin, Asylum Research, (Oxford Instruments, Santa Barbara, CA, USA). Images were acquired in tapping mode using biolever mini cantilevers (AC40TS, Olympus) with nominal spring constant *k* ~ 0.09 N/m). Images were taken in either 512 × 512 or 256 × 256-pixel resolution with a scan rate of 1 Hz. Care was taken to control the tip-sample force to be <100 pN. Prior to image analysis, images were flattened (1st order) to minimize the background. Image roughness analysis, defect detection, and analysis were performed using the built-in analysis software (AR 16.33.234) (Asylum Research, Goleta, CA, USA). The depth and area histograms were prepared using custom software (Igor Pro 6.37, WaveMetrics, Lake Oswego, OR, USA). Histograms were fitted with multiple Gaussians using a non-linear fitting program (MagicPlot: Saint Petersburg, Russia, https://magicplot.com/).

## 3. Results and Discussion

### 3.1. Overview

**Figure 2:**
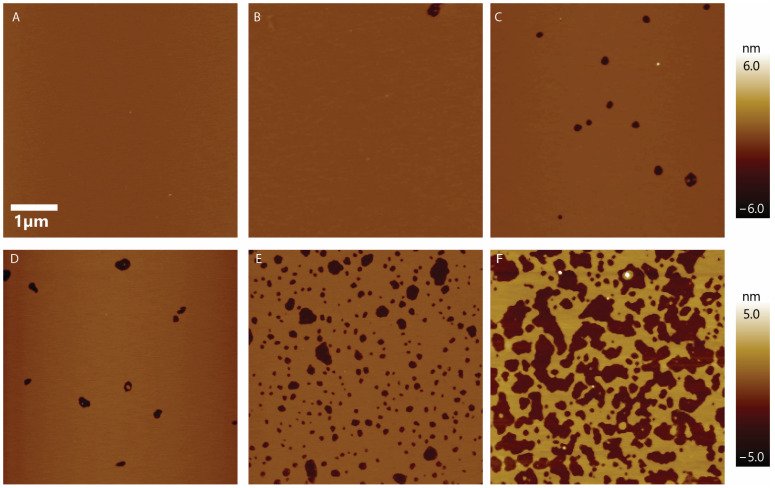
CM15 concentration dependence on defect formation. (**A**–**F**) Empty lipid bilayer, 0.2 μM, 0.5 μM, 1 μM, 2.5 μM, and 5 μM peptide concentration, respectively. When the concentration of peptide was increased, the defects grew larger and, accordingly, the effective roughness of the surface was increased. The 1-micron lateral scale bar applies to all images.

We investigated the topography of the supported lipid bilayer subjected to CM15 at various concentrations. To achieve this, high-resolution in-fluid tapping mode AFM imaging was carried out. We first imaged the bilayer surface that is formed by incubating liposomes on freshly cleaved mica surfaces (Figure 2A). *E. coli* polar extract lipid was used. It contains PE (Phosphatidylethanolamine, 67%), PG (Phosphatidylglycerol, ~23%), and CL (Cardiolipin, ~10%) and closely mimics an inner membrane of bacterial cell membranes [38]. Note that both PG and CL are negatively charged, whereas PE is zwitterionic.

Once the presence of a bilayer was established (see Methods), the desired amount of CM15 was injected into the AFM fluid cell, and images were taken as soon as the instrument settled (about 10 to 15 min). We performed experiments for peptide concentrations ranging from 0.2 µM to 5 µM. Figure 2 shows representative images. Here, we started from a defect-free supported lipid bilayer and increased the peptide concentration in subsequent panels. At 0.2 µM, the effect of CM15 was minimal. As the concentration increased to 0.5 µM, more void-like defects appeared, and the number of voids kept increasing as peptide concentration was increased. At 2.5 µM, numerous punctate pore-like defects were observed. However, at 5 µM, the effect was significantly different; the peptide disrupted the membrane with large defects. For 10 µM, the effect was even larger; the peptide disrupted the bilayer completely (Supporting Information, Figure S4). This clearly demonstrates that CM15 has a significant effect on membrane topography. To confirm that these void-like defects are caused by CM15 and not by AFM probe scanning, we conducted a control experiment where we imaged a supported lipid bilayer in the absence of CM15 for an extended period of time. No significant changes were observed due to AFM probe scanning over the lipid bilayer, even for more than an hour (Figure S1). This confirms that these void-like defects are indeed a result of CM15.

**Figure 3:**
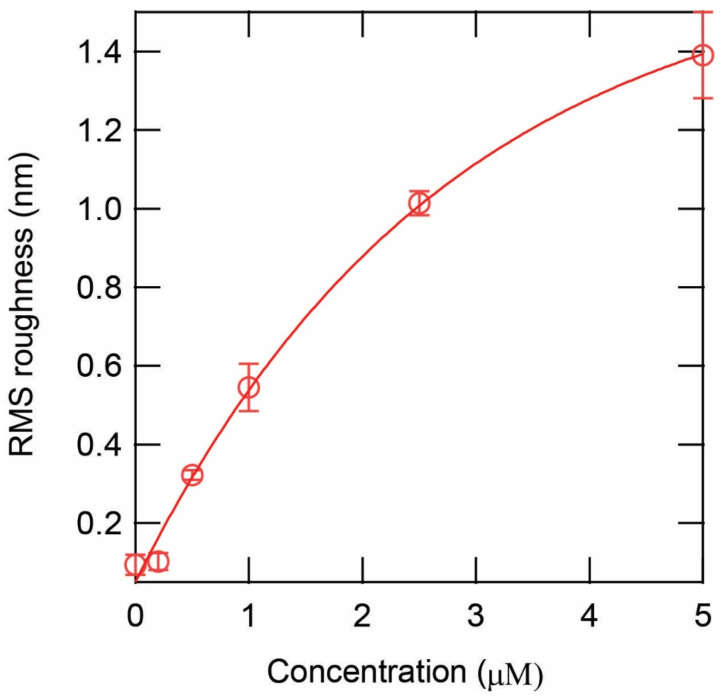
RMS roughness as a function of peptide CM15 concentration. The RMS roughness of the surface after exposure to CM15 peptides.

To quantify the effect, we measured the RMS roughness of the CM15 peptide-treated lipid bilayer surface as a function of peptide concentration. The roughness increased as the concentration of peptide was increased, as shown in Figure 3. The exponential roughness increase was based on measurements from ~ten 5 µm × 5 µm images for each concentration obtained from at least 3 independent experiments. The roughness-increasing effect is consistent with the effect shown by other antimicrobial peptides such as alamethicin and indolicidin [39].

### 3.2. Characterizing the Defects

**Figure 4:**
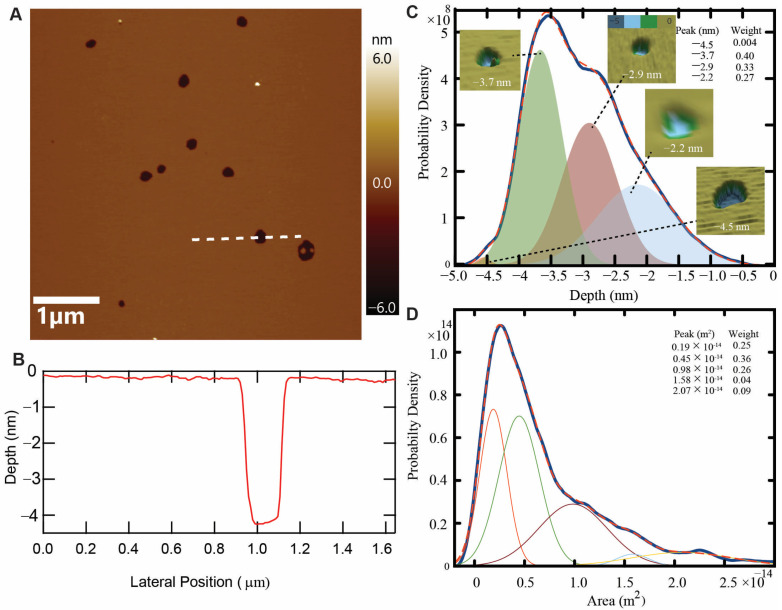
Characterizing defects: (**A**) A representative image of a supported lipid bilayer with 0.5 μM of CM15. (**B**) A depth profile that is taken along the white line shown in (**A**) and demonstrates ~4 nm depth of the defect. (**C**) A smooth histogram of the depth of defects (*N* = 3027) below the lipid bilayer caused by CM15 shows the three major depth populations indicated by shaded gaussians. Note that these histograms include data from peptide concentrations of 0.5, 1, and 2.5 μM. 3D rendering of representative images for each peak, peak value, and their corresponding weightage are also shown. (**D**) Probability density of the areal footprint of the defects shows several populations indicated by different gaussians.

Figure 4A shows a representative image to demonstrate the effect of CM15 at 0.5 µM on a lipid bilayer. Some voids are clearly visible on the membrane surface. Figure 4B shows the depth profile of the image taken at the location of the white dashed line. Here, the zero-depth represents the top surface of the upper leaflet of the untreated bilayer and the deepest position represents the position of the underlying mica surface. To verify if the underlying surface is another lipid bilayer (rather than the underlying solid-state substrate), we analyze the phase image corresponding to Figure 4A (Figure S3). The distinct viscoelasticity apparent in the phase image suggests that the underlying surface is not another bilayer, but a mica surface. The 4 nm depth of the void is similar to the 4 nm characteristic height of the bilayer [40]. This data demonstrates that the void spans the full bilayer, encompassing both bilayer leaflets. To quantify the effect of the peptides in the form of void-like topographical defects, we measured the depth and footprint area of the defects formed by the peptide with concentrations of 0.5, 1, and 2.5 µM and compiled a smoothed histogram, as shown in Figure 4C,D, respectively. Measurements from all the concentrations were compiled together to boost the statistical significance, as the data from each concentration did not exhibit any particular concentration-dependent trend (Figure S3). The depth histogram shows three major topographical defect populations. The major state exhibits a depth of −3.7 ± 0.4 nm (mean ± SD) with an overall probability weight of about 40% and appears to be the most common topographical defect population. The other states, with depths of −2.9 ± 0.5 nm and −2.2 ± 0.7 nm, are present with the respective weights of 33 and 27%. Also, there is a very small number of voids with depths slightly larger than 4 nm. 3D rendering of representative voids for each peak is also shown in Figure 4C. A color scale for the depth is provided at the top of the 2.9 nm feature rendering. We also measured the footprint area of the voids. The resulting area histogram demonstrates several prominent populations, likely related to stable pore geometries. The major area population peaks at 0.45 × 10^−14^ m^2^ and has a weight of about 36%. The other populations are at areas of 0.19 × 10^−14^ m^2^, 0.98 × 10^−14^ m^2^, 1.58 × 10^−14^ m^2^, and 2.07 × 10^−14^ m^2^. Assuming a circular area, the average diameter of the most probable population was calculated to be 76 nm. These voids are larger than the pores formed by the mutated melittin peptide Melp5 in the POPC, having a diameter of about 4 nm [33]. Additionally, these defects are larger than the defects formed by the parent peptide melittin in DOPC bilayers, having a diameter of about 10 nm [41]. This demonstrates that CM15 can form very large void-like defects in *E*. *coli* membranes. The formation of large and stable voids due to CM15 is consistent with previous findings [27]. However, the concurrent formation of transient smaller pores that our force probe would not be able to detect cannot be ruled out. We also plotted the depth as a function of the areal footprint of the void-like defects (Figure S5). Curiously, for many areas, one can find feature depths < 4 nm. These shallow features could correspond to intermediates on the pore formation pathway. The depth of the smallest diameter pores is likely to be limited by the tip geometry.

### 3.3. Formation of Stable Membrane-Spanning Defects and the Resealing of Some Defects

**Figure 5:**
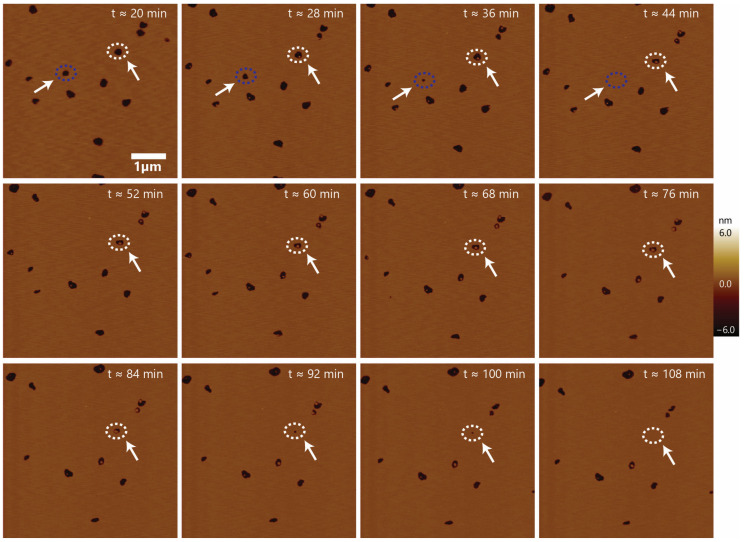
Time-lapse imaging: time-lapse imaging of the defects on the supported lipid bilayer caused by CM15 peptides at 1 μM concentration. The first image was completed ~20 min after the addition of the peptide on the supported lipid bilayer. Most of the defects are stable except some of them are observed to seal up (dashed circle with arrow).

To test the stability of the void-like defects formed by the peptide, we performed time-lapse imaging of a supported lipid bilayer with 1 µM initial CM15 concentration, as shown in Figure 5. Imaging commenced upon peptide injection into the fluid cell, followed by lowering the head of AFM with the force probe onto the sample surface. It requires a certain amount of time (~10–15 min) to position the probe on the sample surface and complete the initial scan. Interactions between the peptide and bilayer occurring in the first several minutes could not be captured because the instrument has to be allowed to settle after condition changes. The t ≈ 20 min in the first image in Figure 5 represents the approximate time that it takes to lower the AFM probe before imaging. Subsequently, images were taken at ~8-min intervals. Void-like defects were observed from the beginning of the imaging sequence (Figure 5, upper left panel). There was no bilayer thinning observed at the beginning of the defect formation unlike the observed bilayer thinning effect caused by other antimicrobial peptides such as MelP5 [33]. From the time-lapse images of the sample over 100 min, it is observed that CM15 causes the formation of very stable defects on the supported lipid bilayer. Most of the observed defects appear to span the depth of the full bilayer, which demonstrates that CM15 is capable of remodeling both leaflets of the bilayer. CM15 was observed to rapidly interact with the bacterial membrane bilayer, which is consistent with previous findings [27]. Some internal structures with a similar height to the bilayer were also observed inside the defects as the imaging continued. After a certain time, some of the voids started sealing. As indicated (Figure 5, arrow and blue circle), a void closed at around 44 min. Also, another void (white dotted circle) closed at over 100 min. No voids were observed to reopen within the imaging timeframe. The presence of voids throughout the time-lapse images demonstrates that CM15 can form defects that are stable on the timescale of >1 h.

**Figure 6:**
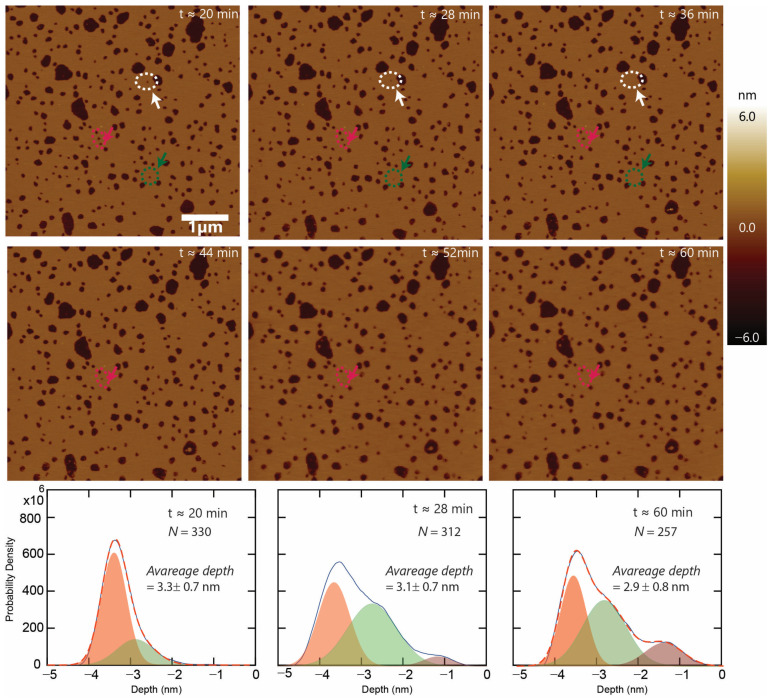
Time-lapse imaging for 2.5 μM peptide: time-lapse imaging of the defects on the supported lipid bilayer caused by CM15 peptides at 2.5 μM concentration. The defects are stable, except some of them are sealing up (please follow the circular marks with an arrow). Lower panel: a smooth histogram of the depth of the voids for an image at t = 20 min, 28 min, and 60 min, respectively. Depth profile evolution can clearly be seen as a function of time. Different populations are indicated by shaded gaussians in each profile. In addition, the number of voids per image and average depth of the voids are also decreasing.

To further investigate the nature of the defects formed by higher peptide concentrations, time-lapse imaging of supported lipid bilayers with 2.5 µM of CM15 was also performed (Figure 6). Similar to the 1 µM case, stable voids were observed at 2.5 µM, as well as voids that appeared to seal up or vanish. Some of the voids closed faster than others. As shown in Figure 6, two voids, circled white and green, and one of the voids, enclosed within the pink ellipse, closed after about 36 min, while another void enclosed inside the pink ellipse took about 52 min to close.

To analyze the behavior of the defects as a function of time, depth histograms for each image time were constructed, three of which are plotted (Figure 6, lower panels). Significant changes in the depth profile of the images as a function of time are clearly noticed. The histogram of the image at t = 20 min indicates only two populations: a major population (weight = 74%) with a depth of 3.5 ± 0.4 nm and a sub-population (weight = 26%) with a depth of 2.9 ± 0.7 nm. In contrast, the image at t = 28 min exhibited 3 distinct populations with depth peaks at 3.6 ± 0.4 nm, 2.7 ± 0.7, and 1.2 ± 0.4 nm, respectively. Interestingly, this three-population behavior extended out to the end of the imaging time course. At t = 60 min, the depth histogram exhibited peaks at identical locations (within uncertainty) to the t = 28 min data; in particular, depth populations of 3.6 ± 0.4 nm, 2.9 ± 0.6 nm, and 1.4 ± 0.5 nm were recorded. An interesting evolution was also observed in terms of the number of voids per image and the average depth of the voids. The number of voids was found to decrease from 330 to 312 to 257 for images at t = 20 min, 28 min, and 60 min, respectively. Similarly, the average depths measured were 3.3 ± 0.7 nm, 3.1 ± 0.7, and 2.9 ± 0.8 at t = 20 min, 28 min, and 60 min, respectively. These observations are consistent with a slow underlying process that caused some voids to reseal or close completely over time, discussed below.

The critical diameter of the voids that were closed within the imaging time frame was determined by analyzing approximately 10 voids. The initial diameters of these closing voids ranged from 25 nm to 190 nm, but the diameter right before closing was found to be 23 ± 7 nm (average ± SD). We note that, due to the finite size of the AFM probe and its convolution with the geometry of the topographical feature to be measured, the absolute values of depth, area, and diameter are not entirely accurate. Tip deconvolution has to be applied to accurately measure topographical features such as the defects seen on the bilayer surface [42]. In light of measurement uncertainties, our finding is in general agreement with previous work on pore formation produced by CM15 [29].

### 3.4. Membrane Disruption

**Figure 7:**
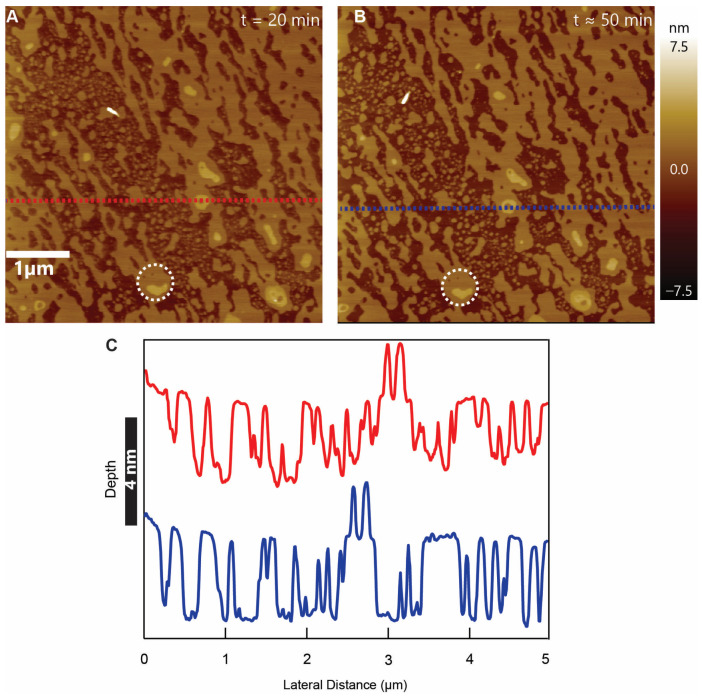
Membrane disruption: (**A**,**B**) Sample images of the defects on the supported lipid bilayer caused by CM15 peptides at 5 μM concentration. CM15 affects the bilayer very quickly, and, thereafter, the defect patterns remain fairly similar. The images show nominally the same areas imaged at the time interval of about 30 min with similar defect patterns. (**C**) Depth profiles taken at the locations of red and blue dotted lines (in (**A**,**B**)) demonstrate similar defect patterns. The lateral displacement between features is due to lateral drift. The black vertical scale represents the scale of the depth profile.

For concentrations higher than 2.5 µM, the systematic void-like defects morphed into large-scale bilayer distortion. For example, the effect of 5 µM CM15 on the supported lipid bilayer is shown in Figure 7. The two images shown were taken at the nominally same location 30 min apart. From the height profiles taken at the locations of the dotted lines in the images and observing individual features (Figure 7, circles), the bilayer was found to be significantly disrupted, and the defect pattern remained stable for at least 30 min, as shown. Additionally, at a concentration of 10 µM, the peptides caused near-complete dissolution of the membrane (Figure S4). The disruption of the membrane with higher concentration is consistent with previous studies. However, previous work using a fluorescent leakage assay showed that CM15 did not cause significant leakage of POPC: POPG (2:1) lipid vesicles at concentrations <10 µM but had an effective leakage at 50 µM. Our study indicates that the leakage could be possible at CM15 concentrations as low as 0.5 µM and should be overwhelming at concentrations >5 µM, as the membrane disruptions occur at higher CM15 concentrations. This discrepancy in the effectiveness of CM15 at a relatively low concentration to disrupt the membrane could be due to the different lipid species that we used and the fact that it is very difficult to quantify peptide-to-lipid (P:L) ratios when supported bilayers are used instead of solution vesicles. Assigning accurate P:L ratios in AFM is challenging because of the requirements of ringing (to prevent loose materials from adhering to the tip) and of a solid-state supporting surface [3]. Clearly, supported lipid bilayers are not able to mix and interact with peptides in the same way as liposomes in solution. In the face of these caveats, we posit that the presence of cardiolipin (CL) in our supported lipid bilayers plays a role in recruiting CM15 to the bilayer. Being a negatively charged lipid, CL enhances the binding of positively charged CM15 with the lipid bilayer and effectively promotes interactions. This is consistent with previous findings where the lytic activity of an AMP was shown to increase in the presence of CL [43].

### 3.5. Defect Dynamics

**Figure 8:**
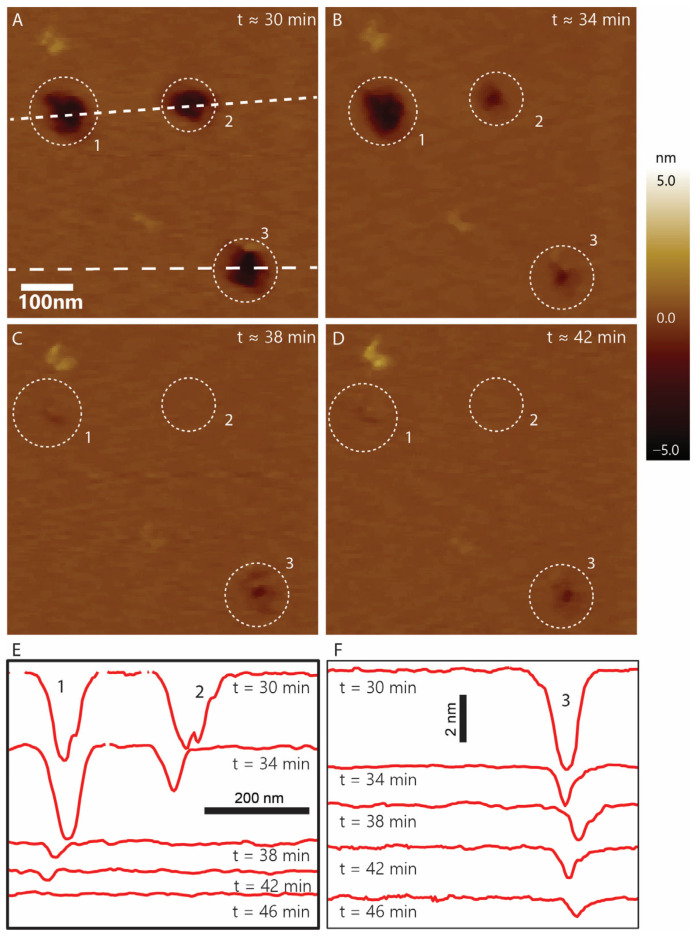
(**A**–**D**) Time-lapse imaging of void-like defects on the supported lipid bilayer caused by CM15 peptides. (**E**,**F**) The depth profile of the feature along the scan line is shown in (**A**). The profiles in (**E**) show the depth profile for features 1 and 2 for each image and (**F**) shows the profile for feature 3.. The times are listed next to each line as well. Two pores close at the end completely.

To analyze the dynamics of these void-like defects, we performed zoomed-in, time-lapse imaging of the defects. As shown in Figure 8, we started with three features labeled 1, 2, and 3. Feature 1 becomes shallower as we continue imaging and closes completely at 46 min as can be seen in the depth profile in Figure 8E (last line). Feature 2 closes completely within 38 min (Figure 8C), while feature 3 does not close completely even after 46 min as indicated by the last depth profile in Figure 8F, though it does significantly reduce in diameter and depth. The filling of the void-like defects and pore closing is consistent with the previous findings for the parent peptide melittin [44], which are well known to form transient pores. Resealing and reappearance of melittin-induced pores have been observed in *E. coli* membranes [44]. It has also been shown that the defects induced by melittin on supported bilayers continuously grow through defect enlargement [41]. Defect sealing, as observed in our study, was also noted in the case of indolicidin when interacting with POPC-supported bilayers at 5 µM peptide concentration [39]. Clearly, further investigation into the mechanism of the resealing effect would be interesting. We note that not all of the voids subject to the same scanning conditions (<100 pN tapping force) were observed to close, but only a subset of pores closed. This further verifies that pore sealing is not due to AFM probe scanning on the bilayer surface.

Our investigation revealed direct visualization of the defects caused by CM15, a hybrid peptide of melittin and cecropin-A. Despite its smaller size compared to both parent peptides, CM15 exhibits remarkable remodeling behavior on a supported lipid bilayer mimicking the bacterial membrane. Previous reports suggested that melittin induces transient membrane leakage for P: L ≤ 1:200 [45]. Equilibrium pores are only observed at higher concentrations. Similarly, cecropin-A causes ion channel formation at lower concentrations (2–5 µM) and membrane destabilization at higher concentrations (>10 µ) [19]. Our study indicates that, despite its smaller size, CM15 can form equilibrium pores in *E. coli* lipid membranes at low concentrations and disruption of membranes at higher concentrations.

Various models have been proposed to describe the mechanism of pore formation by pore-forming peptides. Widely accepted models are barrel-stave, toroidal, and carpet. Melittin has been proposed to follow the toroidal and cecropin-A carpet model, while CM15 has been proposed to follow the toroidal model [10,19,27]. Our data show that CM15 forms robust and stable pores. This is consistent with a toroidal model; however, the AFM resolution is limited. Further work will be required to rule out other models such as the barrel-stave.

## 4. Conclusions

We employed AFM imaging to investigate the lipid bilayer remodeling behavior of CM15 on supported lipid bilayer mimics of the bacterial cell membrane. Our findings indicate that the hybrid peptide CM15 rapidly interacts with the *E. coli* polar lipid bilayer, forming void-like defects spanning both bilayer leaflets. Remodeling effects were observed for CM15 concentrations as low as 0.2 µM, with punctate pore-like defects observed for concentrations up to 2.5 µM. The roughness of the bilayer was found to be increasing as a function of peptide concentration, consistent with the increasing number of peptide-induced membrane defects. Additionally, we observed that some of the voids started self-sealing and eventually vanished completely over time. For higher concentrations of CM15, the defects on the bilayer surface were large-scale and no longer pore-like in nature. Indeed, a complete disruption of the membrane was observed at peptide concentrations of ~10 µM. We posit that the negatively charged lipid headgroups (cardiolipin and PG) enhance the interaction of CM15 and *E. coli* polar bilayers. This work enhances understanding of the mechanism of AMP interactions with supported lipid bilayers.

## Data Availability

The data presented in this study are available within the article itself and in its Supporting Information.

## Supplementary Materials

The following supporting information can be downloaded at: https://www.mdpi.com/article/10.3390/membranes13110864/s1, Figure S1: Time-lapse images on the supported lipid bilayer; Figure S2: Height and phase image comparison; Figure S3: Height and Area histogram for each concentration separately; Figure S4: Disruption of bilayer; Figure S5: Depth vs. Area.

## References

[1] Simon S.A., McIntosh T.J. (2002). Peptide-Lipid Interactions.

[2] Galdiero S., Falanga A., Cantisani M., Vitiello M., Morelli G., Galdiero M. (2013). Peptide-Lipid Interactions: Experiments and Applications. Int. J. Mol. Sci..

[3] Schaefer K.G., Pittman A.E., Barrera F.N., King G.M. (2022). Atomic force microscopy for quantitative understanding of peptide-induced lipid bilayer remodeling. Methods.

[4] Zasloff M. (2002). Antimicrobial peptides of multicellular organisms. Nature.

[5] Faust J.E., Yang P.-Y., Huang H.W. (2017). Action of Antimicrobial Peptides on Bacterial and Lipid Membranes: A Direct Comparison. Biophys. J..

[6] Zhang L., Gallo R.L. (2016). Antimicrobial peptides. Curr. Biol..

[7] Lazzaro B.P., Zasloff M., Rolff J. (2020). Antimicrobial peptides: Application informed by evolution. Science.

[8] Liang Y., Huang Z., Shen X., Zhang Y., Chai Y., Jiang K., Chen Q., Zhao F. (2023). Global Trends in Research of Antimicrobial Peptides for the Treatment of Drug-Resistant Bacteria from 1995 to 2021: A Bibliometric Analysis. Infect. Drug Resist..

[9] White S.H., Wimley W.C. (1998). Hydrophobic interactions of peptides with membrane interfaces. Biochim. Biophys. Acta.

[10] Sabapathy T., Deplazes E., Mancera R.L. (2020). Revisiting the Interaction of Melittin with Phospholipid Bilayers: The Effects of Concentration and Ionic Strength. Int. J. Mol. Sci..

[11] Sato H., Feix J.B. (2006). Peptide–membrane interactions and mechanisms of membrane destruction by amphipathic *α*-helical antimicrobial peptides. Biochim. Biophys. Acta Biomembr..

[12] Wimley W.C. (2018). How Does Melittin Permeabilize Membranes?. Biophys. J..

[13] Chen F.-Y., Lee M.-T., Huang H.W. (2003). Evidence for Membrane Thinning Effect as the Mechanism for Peptide-Induced Pore Formation. Biophys. J..

[14] Dempsey C.E., Sternberg B. (1991). Reversible disc-micellization of dimyristoylphosphatidylcholine bilayers induced by melittin and [Ala-14]melittin. Biochim. Biophys. Acta Biomembr..

[15] Mól A.R., Castro M.S., Fontes W. (2018). NetWheels: A web application to create high quality peptide helical wheel and net projections. BioRxiv.

[16] Andreu D., Ubach J., Boman A., Wåhlin B., Wade D., Merrifield R.B., Boman H.G. (1992). Shortened cecropin A-melittin hybrids. Significant size reduction retains potent antibiotic activity. FEBS Lett..

[17] Juvvadi P., Vunnam S., Merrifield E.L., Boman H.G., Merrifield R.B. (1996). Hydrophobic Effects on Antibacterial and Channel-forming Properties of Cecropin A–Melittin Hybrids. J. Pept. Sci..

[18] Silva T., Claro B., Silva B.F.B., Vale N., Gomes P., Gomes M.S., Funari S.S., Teixeira J., Uhríková D., Bastos M. (2018). Unravelling a Mechanism of Action for a Cecropin A-Melittin Hybrid Antimicrobial Peptide: The Induced Formation of Multilamellar Lipid Stacks. Langmuir.

[19] Efimova S.S., Medvedev R.Y., Chulkov E.G., Schagina L.V., Ostroumova O.S. (2018). Regulation of the Pore-Forming Activity of Cecropin A by Local Anesthetics. Cell Tiss. Biol..

[20] Lee E., Jeong K.-W., Lee J., Shin A., Kim J.-K., Lee J., Lee D.G., Kim Y. (2013). Structure-activity relationships of cecropin-like peptides and their interactions with phospholipid membrane. BMB Rep..

[21] Lyu C., Fang F., Li B. (2019). Anti-Tumor Effects of Melittin and Its Potential Applications in Clinic. Curr. Protein Pept. Sci..

[22] Zhai Z., Zhang F., Cao R., Ni X., Xin Z., Deng J., Wu G., Ren W., Yin Y., Deng B. (2019). Cecropin A Alleviates Inflammation Through Modulating the Gut Microbiota of C57BL/6 Mice With DSS-Induced IBD. Front. Microbiol..

[23] Pistolesi S., Pogni R., Feix J.B. (2007). Membrane Insertion and Bilayer Perturbation by Antimicrobial Peptide CM15. Biophys. J..

[24] Ma L., Luo Y., Ma Y.-H., Lu X. (2021). Interaction between Antimicrobial Peptide CM15 and a Model Cell Membrane Affected by CM15 Terminal Amidation and the Membrane Phase State. Langmuir.

[25] Schlamadinger D.E., Wang Y., McCammon J.A., Kim J.E. (2012). Spectroscopic and Computational Study of Melittin, Cecropin A, and the Hybrid Peptide CM15. J. Phys. Chem. B.

[26] Wang Y., Schlamadinger D.E., Kim J.E., McCammon J.A. (2012). Comparative molecular dynamics simulations of the antimicrobial peptide CM15 in model lipid bilayers. Biochim. Biophys. Acta Biomembr..

[27] Milani A., Benedusi M., Aquila M., Rispoli G. (2009). Pore forming properties of cecropin-melittin hybrid peptide in a natural membrane. Molecules.

[28] Abrunhosa F., Faria S., Gomes P., Tomaz I., Pessoa J.C., Andreu D., Bastos M. (2005). Interaction and Lipid-Induced Conformation of Two Cecropin-Melittin Hybrid Peptides Depend on Peptide and Membrane Composition. J. Phys. Chem. B.

[29] Sato H., Feix J.B. (2006). Osmoprotection of bacterial cells from toxicity caused by antimicrobial hybrid peptide CM15. Biochemistry.

[30] Bhargava K., Feix J.B. (2004). Membrane Binding, Structure, and Localization of Cecropin-Mellitin Hybrid Peptides: A Site-Directed Spin-Labeling Study. Biophys. J..

[31] Chada N., Sigdel K.P., Gari R.R.S., Matin T.R., Randall L.L., King G.M. (2015). Glass is a Viable Substrate for Precision Force Microscopy of Membrane Proteins. Sci. Rep..

[32] Nguyen P.H., Sigdel K.P., Schaefer K.G., Mensah G.A.K., King G.M., Roberts A.G. (2020). The effects of anthracycline drugs on the conformational distribution of mouse P-glycoprotein explains their transport rate differences. Biochem. Pharmacol..

[33] Pittman A.E., Marsh B.P., King G.M. (2018). Conformations and Dynamic Transitions of a Melittin Derivative That Forms Macromolecule-Sized Pores in Lipid Bilayers. Langmuir.

[34] Hammond K., Ryadnov M.G., Hoogenboom B.W. (2021). Atomic force microscopy to elucidate how peptides disrupt membranes. Biochim. Biophys. Acta Biomembr..

[35] Roussel G., Lindner E., White S.H. (2022). Topology of the SecA ATPase Bound to Large Unilamellar Vesicles. J. Mol. Biol..

[36] Sigdel K.P., Wilt L.A., Marsh B.P., Roberts A.G., King G.M. (2018). The conformation and dynamics of P-glycoprotein in a lipid bilayer investigated by atomic force microscopy. Biochem. Pharmacol..

[37] Alessandrini A., Seeger H.M., Caramaschi T., Facci P. (2012). Dynamic Force Spectroscopy on Supported Lipid Bilayers: Effect of Temperature and Sample Preparation. Biophys. J..

[38] Soblosky L., Ramamoorthy A., Chen Z. (2015). Membrane Interaction of Antimicrobial Peptides Using *E. coli* Lipid Extract as Model Bacterial Cell Membranes and SFG Spectroscopy. Chem. Phys. Lipids.

[39] Swana K.W., Nagarajan R., Camesano T.A. (2021). Atomic Force Microscopy to Characterize Antimicrobial Peptide-Induced Defects in Model Supported Lipid Bilayers. Microorganisms.

[40] Lind T.K., Wacklin H., Schiller J., Moulin M., Haertlein M., Pomorski T.G., Cárdenas M. (2015). Formation and Characterization of Supported Lipid Bilayers Composed of Hydrogenated and Deuterated *Escherichia coli* Lipids. PLoS ONE.

[41] Pan J., Khadka N.K. (2016). Kinetic Defects Induced by Melittin in Model Lipid Membranes: A Solution Atomic Force Microscopy Study. J. Phys. Chem. B.

[42] Sanganna Gari R.R., Frey N.C., Mao C., Randall L.L., King G.M. (2013). Dynamic Structure of the Translocon SecYEG in Membrane: Direct Single Molecule Observations. J. Biol. Chem..

[43] Harrison P.L., Heath G.R., Johnson B.R.G., Abdel-Rahman M.A., Strong P.N., Evans S.D., Miller K. (2016). Phospholipid dependent mechanism of smp24, an *α*-helical antimicrobial peptide from scorpion venom. Biochim. Biophys. Acta Biomembr..

[44] Yang Z., Choi H., Weisshaar J.C. (2018). Melittin-Induced Permeabilization, Re-sealing, and Re-permeabilization of *E. coli* Membranes. Biophys. J..

[45] Guha S., Ghimire J., Wu E., Wimley W.C. (2019). Mechanistic Landscape of Membrane-Permeabilizing Peptides. Chem. Rev..

