# Supplementary material for: Effect of CM15 on Supported Lipid Bilayer Probed by Atomic Force Microscopy
Source: Membranes (Basel). 2023 Oct 28;13(11):864. doi: 10.3390/membranes13110864 (PMC10672887; doi:10.3390/membranes13110864)
Supplement: Supplementary file 1 [file membranes-13-00864-s001.zip › membranes-2653777-supplementary.pdf]

Supporting Information

Effect of CM15 on Supported Lipid Bilayer Probed by Atomic Force Microscopy

Olivia D. Walsh, Leona Choi, and Krishna P. Sigdel*

(October 28, 2023)

Department of Physics and Astronomy, California State Polytechnic University, Pomona, CA 91768, USA.

*Corresponding author, emails: kpsigdel@cpp.edu, phone: 909-869-6389

Figure S1 presents time-lapse images of the supported lipid bilayer in the absence of CM15. No significant changes were seen in the images which verifies that the scanning AFM probe does not cause significant changes on the bilayer surfaces. We also presented a punch-through experiment where the AFM probe is forced to punch through the bilayer. This verifies the presence of a bilayer on the mica substrate.

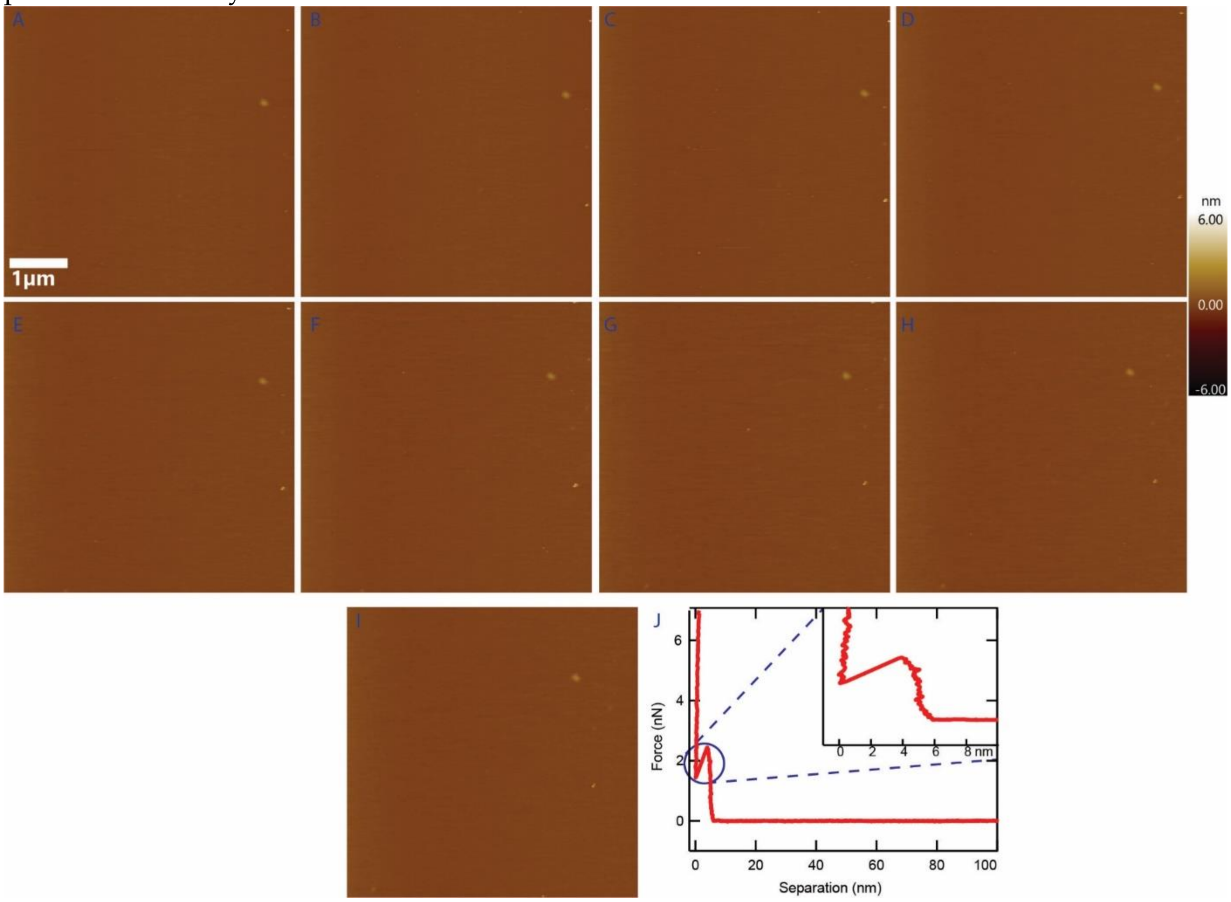

Figure S1: Time-lapse images on the supported lipid bilayer. (A-I) Time lapse images of the empty lipid bilayer taken in the interval of 8.30 minutes. Any significant changes could not be seen on the surface even scanning after an extended period of time. (J) A punch-through curve taken on the surface to confirm the presence of a lipid bilayer. The 4 nm step in the inset clearly shows the evidence of the presence of a bilayer.

Figure S2 compares the same area on the supported lipid bilayer as seen through height imaging (A) as well as phase imaging (B). The different viscoelastic properties of the top part and the underlying surface indicate that the surface underneath the bilayer is different than the surface which confirms that the underlying surface is not another unsupported lipid bilayer and is likely to be the mica substrate.

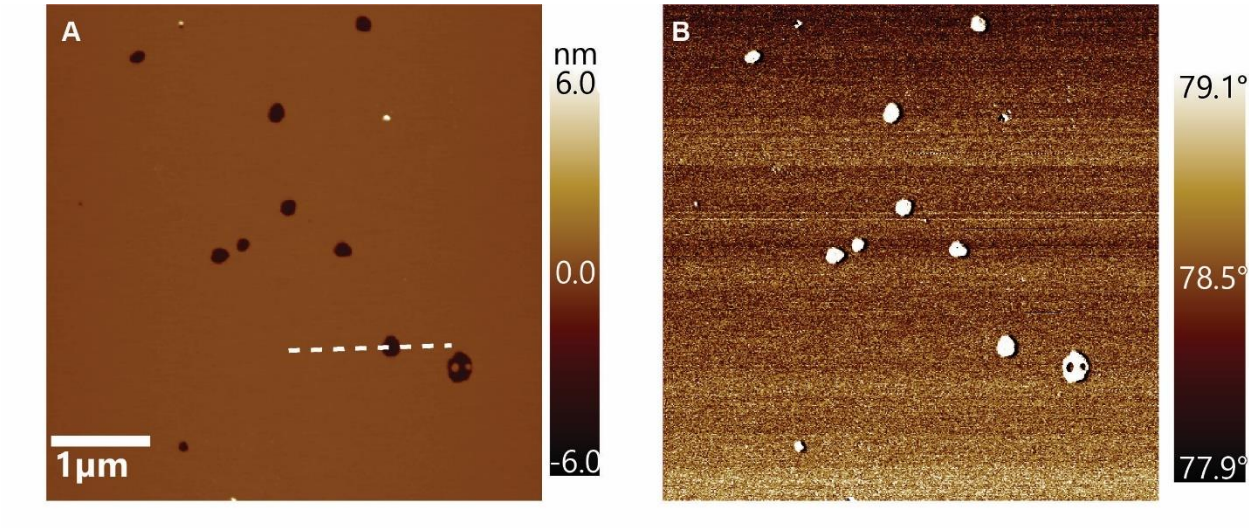

Figure S2: Height and phase image comparison. The comparison of (A) AFM height and (B) phase images of Figure 4 (A). The phase image indicates that the surface underneath the bilayer is not another bilayer and likely to be the mica substrate.

Figure S3 shows the probability density of depth and areal footprint of the defect formed due to CM15 addition on the bilayer. It is hard to see any trend as a function of the peptide concentrations.

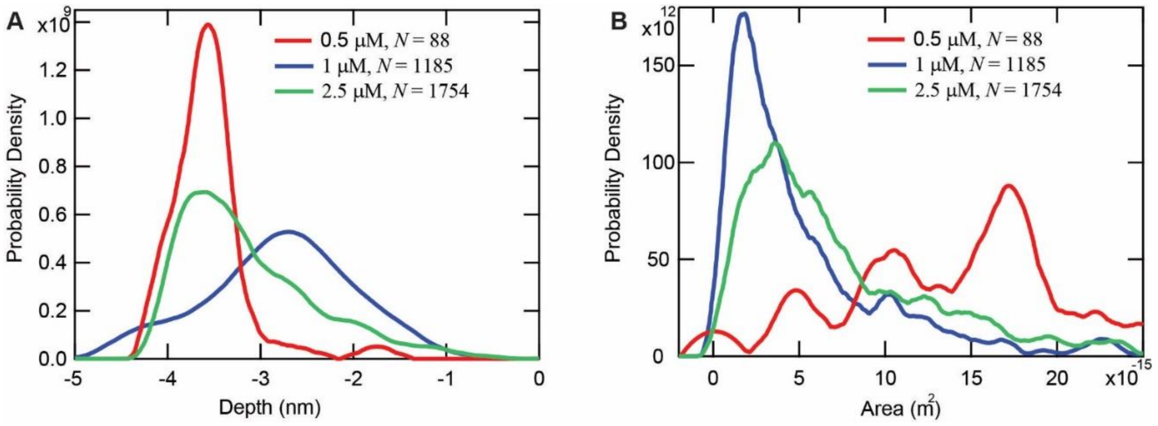

Figure S3: **Height and Area histogram for each concentration separately**. Probability density of (A) Height and (B) areal footprint of defect for individual CM15 concentration. The data does not exhibit any specific peptide concentration-dependent trend.

Figure S4 shows the remodeling effect of the bilayer due to CM15 interaction at 10 μM concentration. This image demonstrates a complete disruption of the bilayer.

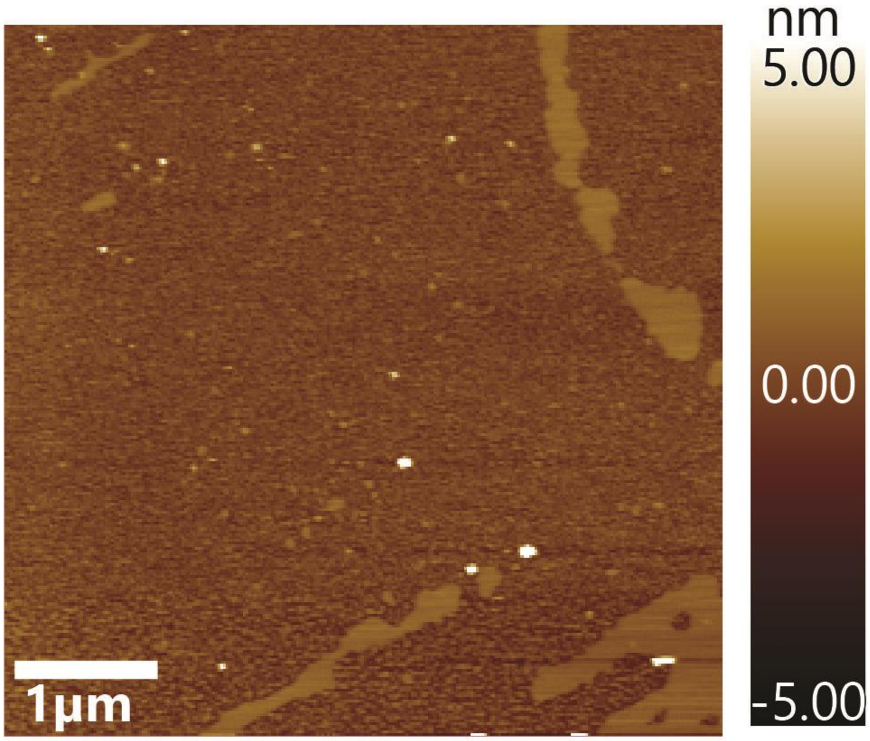

Figure S4: **Disruption of bilayer**: AFM image of supported lipid bilayer completely disrupted by CM15 peptides at 10 μM concentration.

Figure S5 shows the relation between the depth of the void-like defect and their footprint area. Curiously, for many areas, feature depths ranging from 1 to 4 nm are observed. Some of these shallow features could correspond to intermediates on the pathway to becoming stable transmembrane pores. The depth of the smallest diameter pores is likely to be limited by the tip geometry.

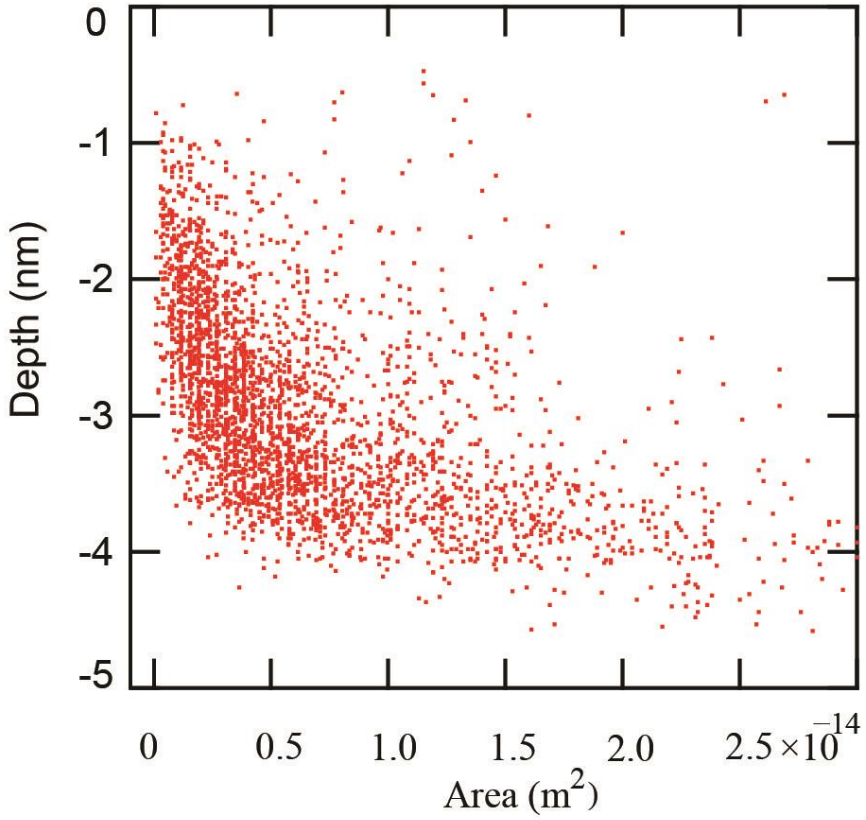

Figure S5: **Depth vs Area**: Depth is plotted as a function of footprint area of the defects caused by CM15 on the supported lipid bilayer.
